# Comprehensive metabolomic profiling in early IgA nephropathy patients reveals urine glycine as a prognostic biomarker

**DOI:** 10.1111/jcmm.16520

**Published:** 2021-05-03

**Authors:** Sehoon Park, Jueun Lee, Seung Hee Yang, Hajeong Lee, Joo Young Kim, Minkyoung Park, Kyu Hong Kim, Jong Joo Moon, Semin Cho, Soojin Lee, Yaerim Kim, Hajeong Lee, Jung Pyo Lee, Chang Wook Jeong, Cheol Kwak, Kwon Wook Joo, Chun Soo Lim, Yon Su Kim, Geum‐Sook Hwang, Dong Ki Kim

**Affiliations:** ^1^ Department of Biomedical Sciences Seoul National University College of Medicine Seoul Korea; ^2^ Department of Internal Medicine Armed Forces Capital Hospital Gyeonggi‐do Korea; ^3^ Integrated Metabolomics Research Group Western Seoul Center Korea Basic Science Institute Seoul Korea; ^4^ Kidney Research Institute Seoul National University Seoul Korea; ^5^ Department of Internal Medicine Seoul National University Hospital Seoul Korea; ^6^ Department of Internal Medicine Keimyung University School of Medicine Daegu Korea; ^7^ Department of Internal Medicine Seoul National University College of Medicine Seoul Korea; ^8^ Department of Internal Medicine Seoul National University Boramae Medical Center Seoul Korea; ^9^ Department of Urology Seoul National University Hospital Seoul Korea; ^10^ Department of Chemistry and Nano Science Ewha Womans University Seoul Korea

**Keywords:** glomerulonephritis, glycine, IgA nephropathy, metabolomics, prognosis

## Abstract

Identification of a urinary metabolite biomarker with diagnostic or prognostic significance for early immunoglobulin A nephropathy (IgAN) is needed. We performed nuclear magnetic resonance‐based metabolomic profiling and identified 26 metabolites in urine samples. We collected urine samples from 201, 77, 47, 36 and 136 patients with IgAN, patients with membranous nephropathy, patients with minimal change disease, patients with lupus nephritis and healthy controls, respectively. We determined whether a metabolite level is associated with the prognosis of IgAN through Cox regression and continuous net reclassification improvement (cNRI). Finally, in vitro experiments with human kidney tubular epithelial cells (hTECs) were performed for experimental validation. As the results, the urinary glycine level was higher in the IgAN group than the control groups. A higher urinary glycine level was associated with lower risk of eGFR 30% decline in IgAN patients. The addition of glycine to a predictive model including clinicopathologic information significantly improved the predictive power for the prognosis of IgAN [cNRI 0.72 (0.28‐0.82)]. In hTECs, the addition of glycine ameliorated inflammatory signals induced by tumour necrosis factor‐α. Our study demonstrates that urinary glycine may have diagnostic and prognostic value for IgAN and indicates that urinary glycine is a protective biomarker for IgAN.

## 1 INTRODUCTION

Immunoglobulin A nephropathy (IgAN) is the most common primary glomerulonephritis and an important cause of end‐stage kidney disease worldwide.^1^, ^2^ The diagnosis of IgAN is morphologically confirmed by pathologic information, and pathologic mechanisms based on immune complex formation have been identified.^3^ However, a disease‐specific biomarker has not yet been actively introduced in clinical practice. As the prognosis of IgAN is heterogeneous among patients, recent studies have attempted to identify predictive measures for the prognosis of IgAN.^4^ Recently, metabolomics has been shown to identify biomarkers in diverse kidney diseases.^5^, ^6^, ^7^, ^8^ The metabolomic profile, which is a consequence of one's genomic‐transcriptomic‐proteomic processes and the environmental influence, may be a potential phenotype that reflects the status of complex kidney diseases. As metabolic biomarkers can be non‐invasively measured in body fluids, in addition to pathologic information obtained by kidney biopsy, researchers have attempted to link kidney diseases and metabolomic findings. Specifically, a prognostic metabolite biomarker would help improve the current prognostic prediction tool for IgAN and suggest metabolic pathways that may be potential therapeutic targets for this disease.

In this study, we aimed to investigate a disease‐related biomarker that could be non‐invasively collected in the urine of IgAN patients without reduced kidney function using metabolomic profiling. We first hypothesized that a differentially expressed urinary metabolite biomarker in individuals with early IgAN compared to controls could be identified. Further, we asked whether this biomarker has prognostic value and performed experimental validation to determine the pathophysiologic role of the identified metabolite.

## 2 MATERIALS AND METHODS

### 2.1 Ethical considerations

This study was conducted according to the Declaration of Helsinki. The institutional review board of Seoul National University Hospital, Seoul, Korea (H‐1601‐076‐732) approved the study. All clinical characteristics and biospecimens were prospectively collected with the approval of the study patients with informed consents.

### 2.2 Study population and clinical characteristics

The kidney biopsies and urine sample collection at the timing of diagnosis were performed between October 2009 and March 2016. Patients with pathologically confirmed IgAN from kidney biopsies were included as the study group. We included patients diagnosed with membranous nephropathy (MN), minimal change disease (MCD) or lupus nephritis (LN) as the disease‐control group. We also constructed a healthy control group, with no evidence of kidney dysfunction, including abnormal urinalysis or reduced estimated glomerular filtration rate (eGFR), and assessed donated urine and blood samples from health screening examinees. The baseline clinical characteristics and biospecimens, including morning random urine samples, were collected at the time of kidney biopsy for initial diagnosis and for health screening examinees at their time of visit. eGFR was calculated with the chronic kidney disease epidemiology collaboration equation.^9^ We excluded those with a follow‐up duration less than 3 months or eGFR < 60 mL/min/1.73 m^2^ as we intended to study patients without substantial impairment in their kidney function. In addition, a few prominent outliers regarding urine metabolomic profiles, when identified by visualized principal component analysis plots based on the first and second principal components, were excluded to prevent technical bias. As a clinical outcome, an 30% reduction in eGFR from baseline was used, as most patients had relatively preserved kidney function, and the follow‐up duration was relatively short (median 1.6 years).^10^ The other details regarding the collected variables are described in Supplemental Methods.

### 2.3 Urine metabolomic profiling

The nuclear magnetic resonance (NMR)‐based urine metabolomic profiling method has been previously published, and details are explained in the Supplemental Methods, Figures S1 and S2.^8^

### 2.4 Statistical analysis

During statistical analysis, a biostatistician was blinded to all names of the metabolites, and the names were revealed at the time of planning further experimental validation to limit confirmation bias.

The principal component analysis and partial least squares discriminants analysis were performed by ‘mixOmics’ package in R.^11^

We compared the urinary metabolite levels between the IgAN group and another disease or healthy control group with the Mann‐Whitney *U* test. The correlations between the clinical characteristics and the urinary metabolite levels were investigated with Spearman's test.

The predictability for IgAN was assessed by area under curve analysed by receiver operating characteristics (ROC) curve, and we compared the area under curve by DeLong's method with ‘pROC’ package in R.^12^ The base clinical model included age, sex, baseline MAP, urine protein‐to‐creatinine ratio and eGFR values. The predictability of the model including urine glycine level in addition to the clinical variables was compared to those of the base model and a model including all other urine metabolite levels but urine glycine.

The association between the metabolite values and the risk of poor kidney prognosis, a 30% reduction in eGFR, was determined with panelized spline regression. For those metabolites that had a potential linear association with adverse kidney outcome risks, we performed Cox regression analysis to confirm the prognostic significance. Multivariable models were adjusted for age, sex, baseline eGFR, random urine protein/creatinine ratio, mean arterial pressure and pathologic parameters including the Oxford classification scores, history of angiotensin‐converting enzyme inhibitor or angiotensin II receptor blocker use, and history of immunosuppressive agent use at the time of kidney biopsy to include the variables for the current risk prediction tool for IgAN. We also included sex and crescent scores, which were not included in the suggested prediction tool but might have clinical importance, in the full model.

We tested whether the addition of metabolite levels to a prediction model including clinical information improved the predictability analysed by continuous net reclassification improvement (cNRI) with thousands of bootstrapping cycles.^13^, ^14^ When 95% confidence intervals were above zero, a new model was considered to have better prognostic predictability than a base model. We also asked whether the addition of metabolite levels to a clinical model without pathologic information showed comparable predictability to that of a full prediction model including clinical and pathologic characteristics. Finally, we investigated whether the addition of the identified metabolite improved the predictability of the full model, including both clinical and pathologic characteristics.

In the cNRI analysis, when 95% confidence intervals crossed zero, the difference between the prognostic predictability of the models was interpreted to be non‐significant. A Bonferroni‐adjusted *P* value (0.05/number of comparison) was implemented when identifying differences in urine metabolite levels among the study groups or identifying correlation between the metabolite levels and clinical characteristics. In the prognostic analysis or when comparing model predictive power, conventional two‐sided *P* value < .05 indicated statistical significance. All statistical analyses were performed with R (version 4.0.2, the R foundation).

### 2.5 Immunohistochemistry

For immunohistochemistry, we included pathologic slides from kidney biopsies of 12 IgAN cases, 6 controls and 3 MCD and MN cases. The primary antibodies used for staining were for the glycine cleavage system, including T protein, P protein, L protein, and H protein and SHMT 1 and 2 (Abcam). For staining, we cut unstained, paraffin‐embedded tissue sections that were stored at the time of kidney biopsy into 4 μm sections. The sections were deparaffinized in xylene and rehydrated with a descending concentration series of ethanol. Sliced specimens were heated three times in a microwave oven for 5 minutes with 10% citrate buffer solution (pH 6.0) to retrieve the antigen. We blocked the endogenous streptavidin activity with 3% hydrogen peroxide in methanol for 10 minutes at room temperature. Blocking reagent (Santa Clara, CA, USA) was used to block non‐specific binding. Images were captured using a light microscope system (Leica Microsystems, Germany). Quantification of the immunohistochemical staining was performed with ImageJ (version 1.8.0., National Institute of Health, USA). We measured the proportion of the stained area with the same cut‐off thresholds for at least 5 high‐power fields in each sample, and the average value was used to represent the degree of staining of the sample.

### 2.6 In vitro study

The unaffected kidney cortices of renal cell carcinoma patients were mechanically dissected. In the dissected cortex, the specimens were minced and digested with Hank's 201 balanced salt solution (HBSS) containing 3 mg/mL collagenase (Sigma‐Aldrich, St. Louis, MO, USA) and incubated at 37°C for 1 hour. The digested cells were washed through a series of sieves (120, 70 and 40 μm in diameter) with phosphate‐buffered saline, followed by centrifugation (500 *g* for 5 minutes). The recovered hTECs were collected from the pallet and incubated in DMEM/F12 (Lonza, Basel, Switzerland) for 4 hours. Floating tubules in the media were retrieved and cultured on collagen‐coated petri dishes (BD Biosciences, Franklin Lakes, NJ, USA) until the establishment of colonies of hTECs in REGM medium (Clonetics, CA, USA) supplemented with 10% foetal bovine serum, 12 mg/mL bovine brain extract, 1 mg/mL hydrocortisone, 10 ng/mL epidermal growth factor, 50 mg/mL gentamycin and 50 ng/mL amphotericin B. hTECs at 2‐3 passages were used in the current study.

We first tested the expression of target glycine cleavage system molecules identified by the above immunohistochemistry after different dosages of tumour necrosis factor‐α (TNF‐α). The use of TNF‐α to trigger the inflammatory response in hTECs was based on a previous study regarding the tubule pathophysiology of IgAN. After 1 hour of treatment, hTECs were incrementally treated with 2.5, 5.0, 10.0 and 20.0 ng/mL TNF‐α. The dosage resulting in the expression of the target glycine cleavage system molecule (10 ng/mL for 1 hour) was implemented to stimulate hTECs in the following experiments.

Second, we treated hTECs with 10 ng/mL TNF‐α for 1 hour, with cotreatment of glycine (Merck, NJ, USA) in cell culture media with incremental dosages of 2.5, 5.0, 10.0 and 20.0 mmol/L. We investigated the relative expression of inflammation‐related molecules, including intercellular adhesion molecule 1 (ICAM1), p38, phosphor‐p38, p65 and phosphor‐p65, to β‐actin. We also measured glutathione concentrations using a glutathione assay kit (Promega, WI, USA), along with the inflammation‐related molecules.

Third, we inhibited the target glycine cleavage system molecule (protein H) by siRNA and tested the expression of inflammatory molecules under conditions of TNF‐α induction in hTECs. We transfected the hTECs with siRNA for protein H (Thermo Fischer Scientific, MA, USA) according to the manufacturer's protocol. As controls, hTECs treated with scrambled siRNA and hTECs in conditions without TNF‐α induction were tested. Levels of glycine in cell lysates were measured in each condition by the glycine assay kit (Abcam) with mean fluorescence intensity units.

For immunoblotting of the inflammatory molecules and glycine cleavage system, 80 μg of proteins was separated by electrophoresis on 10% sodium dodecyl sulphate‐polyacrylamide gels and transferred onto Immobilon‐FL 0.4 μmol/L polyvinylidene difluoride membranes (Millipore, Bedford, MA, USA). Proteins were probed with primary antibodies targeting ICAM1 (Thermo Fisher Scientific), p38 (Abcam), phosphor‐p38, p65, phosphor‐p65 (Cell Signaling Technology, MA, USA), protein H and protein T (Abcam) followed by anti‐rabbit IgG (Cell Signaling Technology) secondary antibodies. The labelled proteins were detected by an enhanced chemiluminescence system (ECLTM PRN 2106; Amersham Pharmacia Biotech, Buckinghamshire, UK). Quantification of Western blot bands was performed with ImageJ.

## 3 RESULTS

### 3.1 Study population

We applied ^1^H NMR‐based metabolomic profiling to identify and quantify urinary metabolites in 203 IgAN patients with eGFR ≥ 60 mL/min/1.73 m^2^. With the same criteria applied, MN patients (n = 81), MCD (n = 49), LN (n = 38) patients and healthy controls (n = 146) were profiled for their urine metabolomics. After we excluded the outliers, 201, 77, 47, 36 and 136 patients remained in the IgAN, MN, MCD, LN and healthy control groups, respectively. The baseline clinical characteristics among the study population are shown in Table 1, and the IgAN group had the youngest age distribution, with a median age of 37 years old. All the study patients had eGFR ≥ 60 mL/min/1.73 m^2^, and the MN and MCD groups had higher urinary random protein/creatinine ratios than the other groups. Within the IgAN groups, 170 (85.4%) patients were on angiotensin‐converting enzyme inhibitors or angiotensin II receptor blockers, and 54 (27.1%) patients were on immunosuppressive drugs.

**TABLE 1:** Baseline characteristics of the study population

	IgAN (N = 201)	MN (N = 77)	MCD (N = 47)	LN (N = 36)	Control (N = 136)
Age (years)	37.0 [27.0;48.0]	55.0 [47.0;66.0]	43.0 [31.0;57.5]	30.0 [25.0;36.0]	50.5 [33.5;62.5]
Male sex	99 (49.3%)	27 (35.1%)	13 (27.7%)	29 (80.6%)	52 (38.2%)
Mean arterial pressure (mm Hg)	85.0 [78.0;93.0]	87.0 [80.0;93.0]	90.0 [79.5;96.5]	93.0 [84.0;98.5]	‐
Laboratory findings					
eGFR (mL/min/1.73 m^2^)	97.9 [78.3;115.4]	95.6 [88.1;103.7]	101.1 [90.2;113.6]	115.3 [86.5;125.7]	98.3 [87.0;105.8]
Urine PCR (g/g)	1.0 [0.5; 1.6]	3.8 [2.0; 7.3]	6.3 [2.9; 9.9]	2.6 [1.1; 4.1]	‐
Haemoglobin (g/dL)	12.2 [11.2;13.8]	12.6 [11.4;13.9]	13.3 [12.9;14.5]	10.7 [9.6;11.6]	14.6 [13.6;15.4]
Total cholesterol (mg/dL)	187.0 [165.0;213.0]	236.0 [190.0;278.0]	343.0 [285.0;446.0]	213.5 [186.5;253.5]	192.5 [169.5;216.5]
Uric acid (mg/dL)	5.7 [4.7; 6.8]	6.1 [5.2; 7.0]	6.0 [5.1; 7.7]	5.7 [4.8; 7.1]	5.7 [4.9; 6.4]
Albumin (g/dL)	4.0 [3.7; 4.2]	2.8 [2.2; 3.3]	2.1 [1.8; 2.6]	3.0 [2.7; 3.5]	4.5 [4.2; 4.6]
Pathologic findings					
Glomerular changes					
Global sclerosis (%)	10.0 [3.1;21.1]	6.1 [0.9;11.6]	0.0 [0.0;11.1]	0.0 [0.0; 7.8]	‐
Segmental sclerosis (%)	3.6 [0.0; 9.5]	0.0 [0.0; 0.0]	0.0 [0.0; 0.0]	0.0 [0.0; 5.0]	‐
Crescent formation (%)	0.0 [0.0; 0.0]	0.0 [0.0; 0.0]	0.0 [0.0; 0.0]	0.0 [0.0; 4.6]	‐
Oxford classification					
M1	62 (31.2%)	‐	‐		‐
E1	25 (12.6%)	‐	‐		‐
S1	193 (51.8%)	‐	‐		‐
T1	21 (10.6%)				
T2	2 (1.0%)	‐	‐		‐
C1	21 (10.6%)	‐	‐		‐
C2	1 (0.5%)	‐	‐		‐

Note

Categorical variables are presented as number (%), and continuous variables are shown as median [interquartile ranges].

Abbreviations: eGFR, estimated glomerular filtration rate; IgAN, immunoglobulin A nephropathy; LN, lupus nephritis; MCD, minimal change disease; MN, membranous nephropathy; PCR, protein‐to‐creatinine ratio.

### 3.2 Urinary metabolic profiles

Unsupervised principal component analysis indicated the composite of metabolites did not clearly separate the study groups in total (Figure S3). When we performed supervised partial least squares discriminants analysis, the composite of metabolites showed some discrimination for the healthy control group and there was a large overlap between the other diseased patients.

**FIGURE 1:**
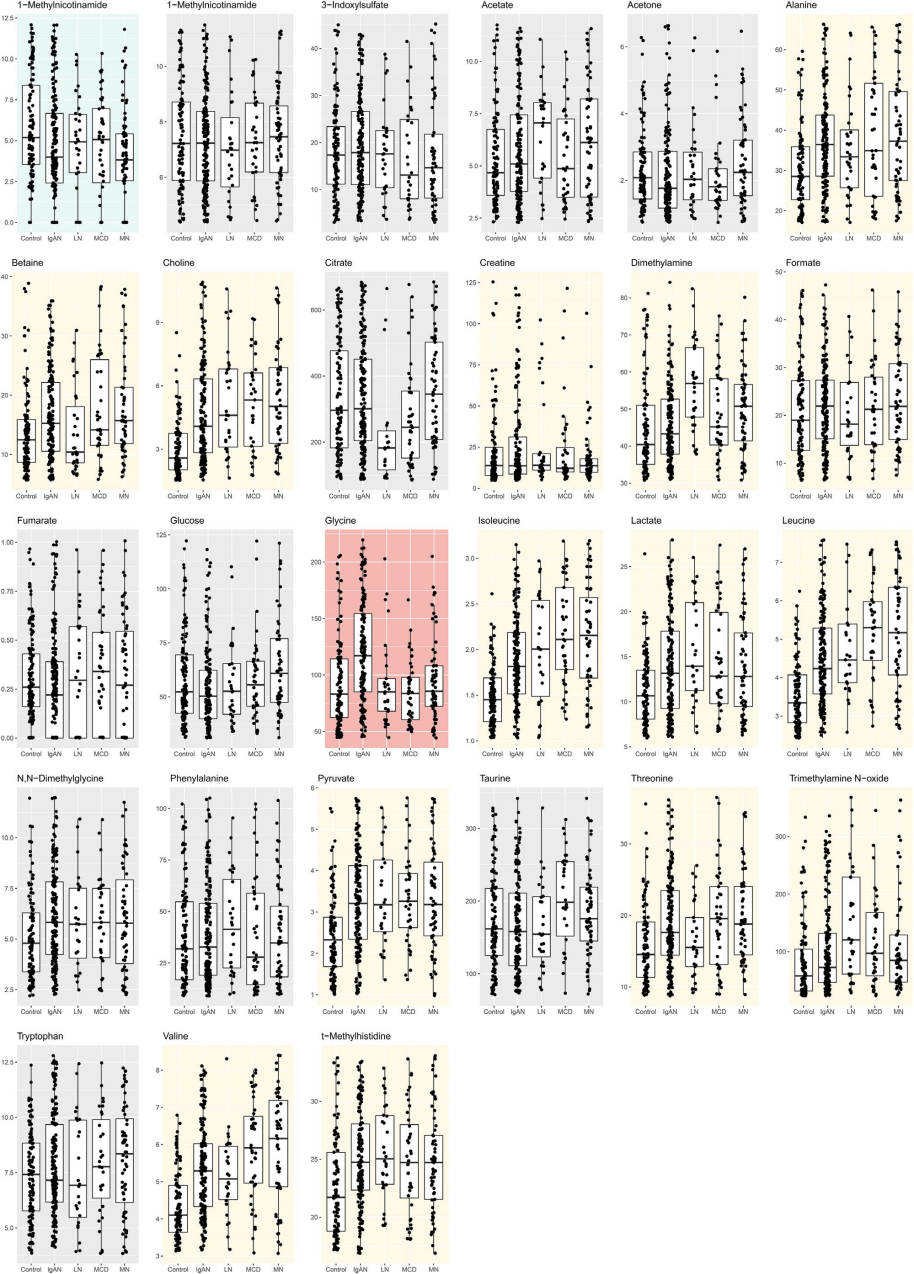
Box plots showing the creatinine‐adjusted urinary metabolite concentrations. The *y*‐axes indicate the creatinine‐adjusted urine concentrations (μmol/L/mmol/L Cr). The box plot shows the interquartile ranges, with the horizontal lines indicating the median values. Each dot represents the value of each study patient, and outliers over 95 percentile ranges are not presented in the figure. The blue background indicates the metabolite that was significantly low in the IgAN samples, and the yellow backgrounds indicate the metabolites that are significantly high in the urine of IgAN patients. The red background indicates that the metabolite level was significantly higher in the IgAN samples than in the disease‐control samples

When individual metabolite level was considered (Figure 1), fifteen metabolites—alanine, betaine, choline, creatinine, dimethylamine, formate, glycine, isoleucine, lactate, leucine, pyruvate, threonine, trimethylamine N‐oxide, valine and t‐methylhistidine—were significantly higher in the IgAN group than in the healthy control group (Figure 1 and Table S1). However, most of the metabolites were similarly increased in the disease‐control groups compared to the healthy control group, and only the glycine levels were significantly higher in the IgAN patients than in the MCD (*P* < .001), MN (*P* = .001) and LN (*P* = .001) patients. The glycine level was higher in the MN cases than in the controls, but the significance did not reach the Bonferroni‐adjusted significance level (*P* = .017), and the MCD (*P* = .584) or LN group (*P* = .798) showed similar levels as the healthy control group.

When area under curve of ROC curve by logistic regression model predicting IgAN was calculated, the model including urine glycine level along with clinical variables showed significantly higher area under curve than the base clinical model including age, sex, urine protein‐to‐creatinine ratio, eGFR and mean arterial pressure (Table 2). When the predictability of the model including urine glycine level was compared to that of the model including all other urine metabolites but glycine, the difference in area under curve was non‐significant. The model including all urine metabolite levels including urine glycine showed the highest area under curve than the other constructed models.

**TABLE 2:** Predictability of IgAN assessed by area under curve of the ROC curve analysis

Variables included in model	Area under curve (95% CI)	*P* value for difference in area under curve with the model of interest^†^
Age, sex, baseline eGFR, mean arterial pressure, urine PCR and urine glycine level (model of interest)	0.909 (0.877‐0.941)	NA^‡^
Age, sex, baseline eGFR, mean arterial pressure, urine PCR	0.884 (0.848‐0.921)	.026
Age, sex, baseline eGFR, mean arterial pressure, urine PCR and all measured urine metabolites except for glycine	0.916 (0.886‐0.946)	.417
Age, sex, baseline eGFR, mean arterial pressure, urine PCR and all measured urine metabolites including glycine	0.931 (0.903‐0.958)	.006

Abbreviations: CI, confidence interval; ROC, receiver operating characteristics.

^†^ *P* value was calculated by Delong's method.

^‡^ *P* value for difference in area under curve was not calculated as this was the model of interest.

### 3.3 Urinary metabolites associated with the clinical characteristics and prognosis of IgAN

When we tested the correlations between the urinary metabolite levels and the clinical characteristics of the IgAN patients, several positive associations were revealed between the urine protein‐to‐creatinine ratio and several metabolites (Figure S4). Urine glucose level was correlated with diabetes mellitus, reaching Bonferroni‐corrected significance level. In addition, some metabolites were associated with older ages; however, glycine did not show a Bonferroni‐corrected significant level correlation with the other clinical characteristics.

**FIGURE 2:**
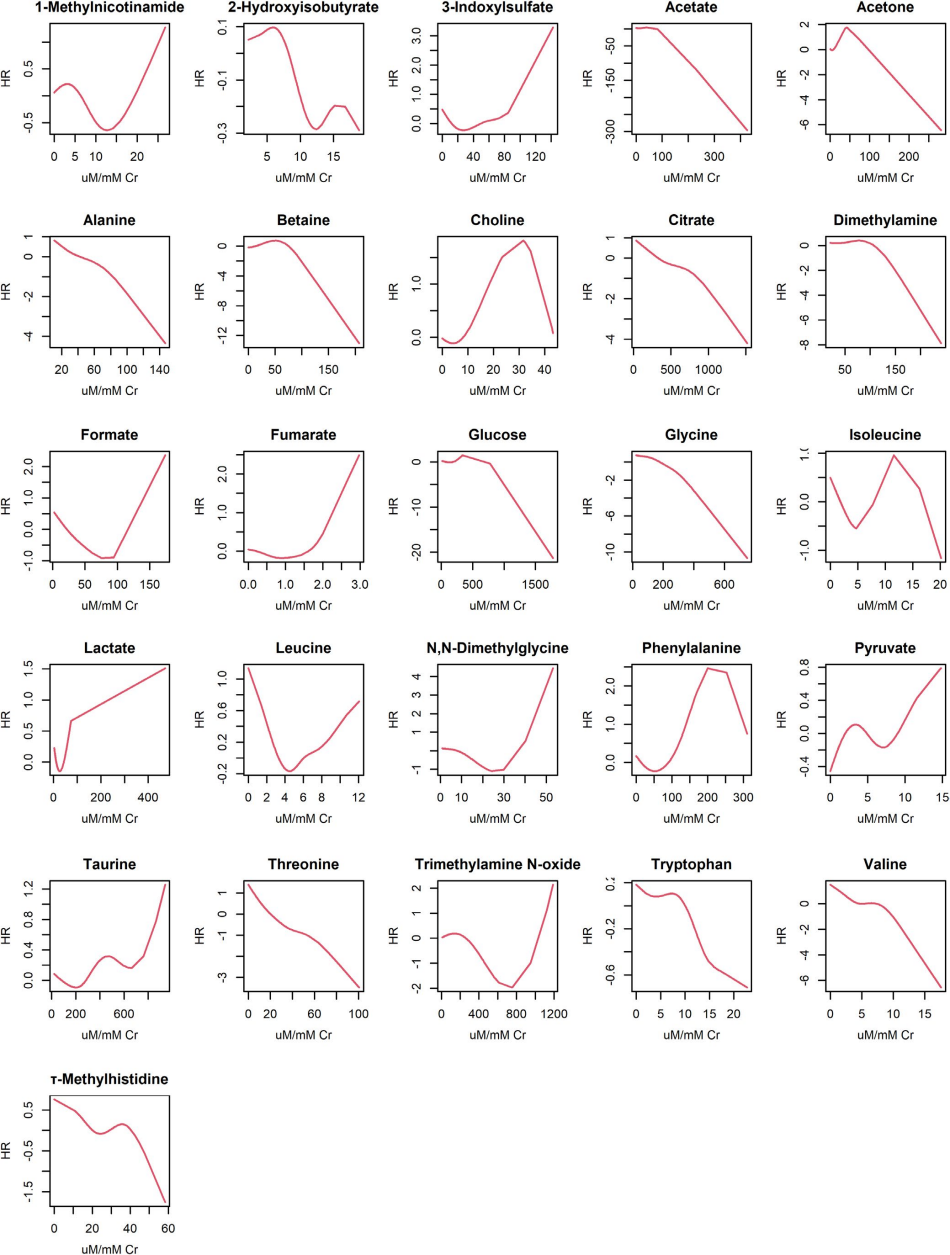
Association between the creatinine‐adjusted urinary metabolite concentrations and the risk of a 30% reduction in eGFR. The *y*‐axes indicate the unadjusted hazard ratios for risk of a 30% reduction in eGFR. The *x*‐axes indicate the creatinine‐adjusted urine metabolite levels. The red graphs indicate the associations between the metabolite levels and the risk of the study outcome

Next, we asked whether certain urinary metabolites were linearly associated with the risk of adverse renal outcomes with panelized spline curves. Several metabolites showed a negative association with the risk of a 30% reduction in eGFR (Figure 2). The associations between the metabolite levels and the risk of a 30% reduction in eGFR remained significant in the multivariable analysis with alanine, glycine, threonine and valine (Table S2). When we tested the correlations of urinary metabolite levels, we identified certain correlations with urine metabolite levels. In particular, significant correlations between the levels of alanine, glycine, threonine and valine that showed prognostic significance were found, (Figure S5).

When we tested the additive prognostic value of the identified metabolites, the addition of glycine or other metabolites to a clinical model improved the prognostic predictability (Table 3).^4^ In addition, a clinical model including information on a metabolite but without pathologic information showed non‐inferior predictability to the full model, which included both clinical and pathologic characteristics. Furthermore, the addition of glycine or other urinary metabolites improved the predictability of the full model, which included age, sex, MAP, proteinuria, MEST‐C scores, history of angiotensin‐converting enzyme inhibitor or angiotensin II receptor blocker use, and history of immunosuppressive agent use.

**TABLE 3:** The discrimination performance of models predicting the eGFR 30% reduction risk within 3 y post‐biopsy

Comparison	Models	cNRI (95% CI)^§^	C‐index (standard error)
Clinical parameters^†^ vs clinical parameters with metabolite	Clinical parameters	‐	0.730 (0.046)
	Clinical parameters + alanine	0.73 (0.21‐1.09)	0.741 (0.043)
	Clinical parameters + glycine	0.72 (0.28‐0.82)	0.751 (0.046)
	Clinical parameters + threonine	0.83 (0.26‐1.20)	0.739 (0.044)
	Clinical parameters + valine	0.65 (0.14‐0.99)	0.737 (0.046)
Clinical parameters and pathologic information^‡^ vs clinical parameters without pathologic information but with metabolite	Clinical parameters + pathologic information	‐	0.742 (0.047)
	Clinical parameters + alanine	‐0.02 (−0.82‐0.74)	0.741 (0.043)
	Clinical parameters + glycine	0.29 (−0.92‐0.66)	0.751 (0.046)
	Clinical parameters + threonine	0.43 (−0.72‐0.69)	0.739 (0.044)
	Clinical parameters + valine	‐0.01 (−0.69‐0.56)	0.737 (0.046)
Clinical parameters and pathologic information^‡^ vs clinical parameters and pathologic information with metabolite	Clinical parameters + pathologic information	‐	0.742 (0.047)
	Clinical parameters + pathologic information + alanine	0.60 (0.04‐1.08)	0.759 (0.041)
	Clinical parameters + pathologic information + glycine	0.60 (0.05‐1.23)	0.757 (0.044)
	Clinical parameters + pathologic information + threonine	0.81 (0.16‐1.29)	0.757 (0.046)
	Clinical parameters + pathologic information + valine	0.71 (0.02‐1.27)	0.763 (0.043)

Abbreviations: CI, confidence interval; cNRI, continuous net reclassification improvement.

^†^ The clinical model without pathologic information included following variables: age, sex, baseline eGFR, mean arterial pressure, urine protein‐to‐creatinine ratio, history of angiotensin‐converting enzyme inhibitor or angiotensin II receptor blocker, and history of immunosuppressive agent at the time of kidney biopsy.

^‡^ The full multivariable model included age, sex, baseline eGFR, mean arterial pressure, urine protein‐to‐creatinine ratio, MEST‐C pathologic scores, history of angiotensin‐converting enzyme inhibitor or angiotensin II receptor blocker, and history of immunosuppressive agent at the time of kidney biopsy. The analysis was performed with 191 IgAN patients with non‐missing information in the covariates.

^§^ When the 95% CI was greater than zero, the model has better prognostic predictability than the base model. When the 95% CI crosses zero, the difference in prognostic predictability between the models is non‐significant.

### 3.4 Validation of glycine metabolites by quantitative LC‐MS analysis

Among the metabolites associated with the prognosis of IgAN, glycine was selected as our target urine metabolite for further experimental validation because only glycine was significantly increased in the urine from the IgAN group compared to the disease‐control group. Additionally, this metabolite has been suggested to be associated with a lower risk of incident chronic kidney disease.^15^ We confirmed that the urinary glycine levels measured by NMR analysis were highly correlated with those measured by LC‐MS analysis with absolute quantification (Figure S6).

### 3.5 Immunohistochemistry results

**FIGURE 3:**
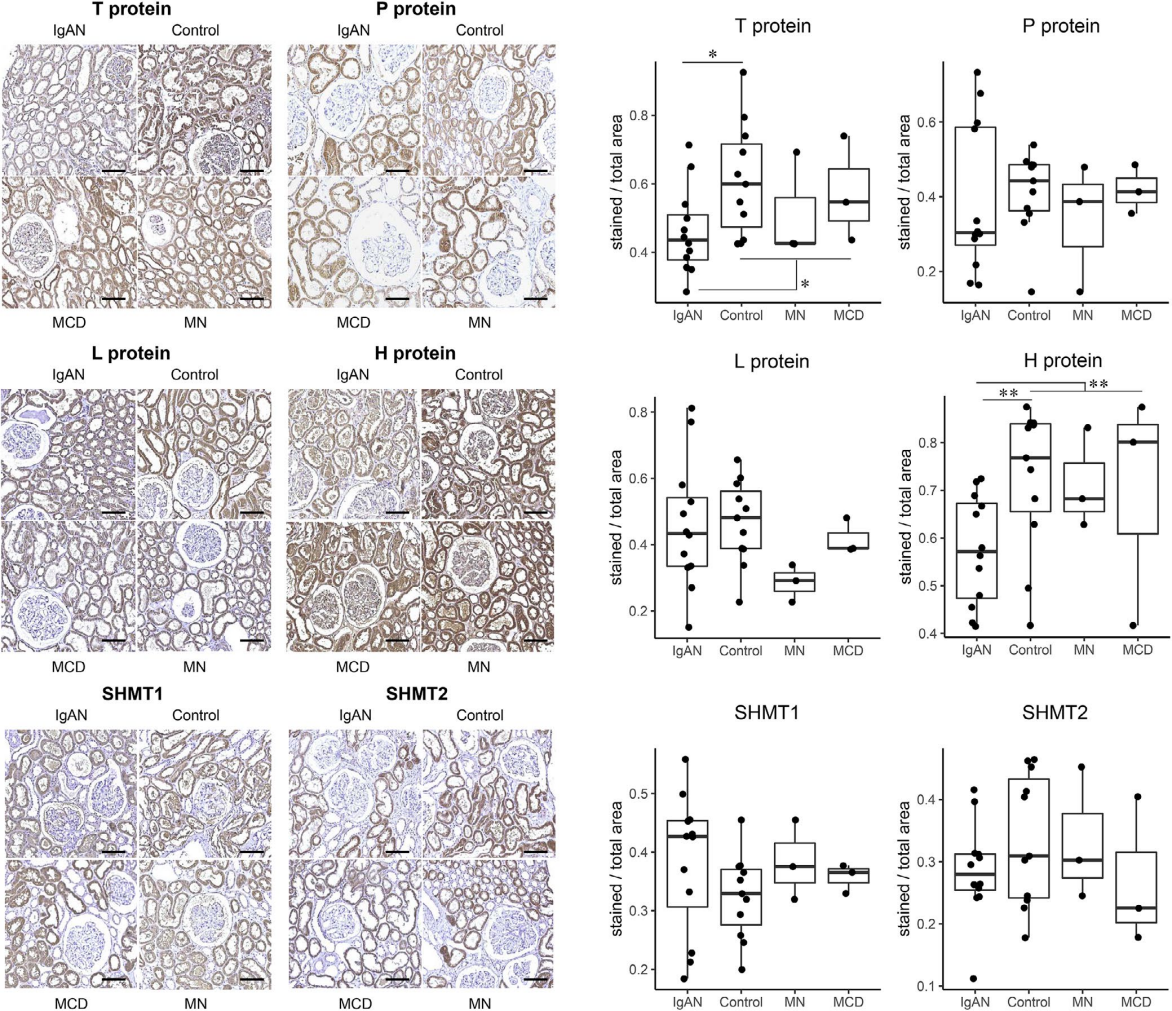
Representative immunohistochemistry results for the enzymes related to glycine metabolism. Representative immunohistochemistry images of human kidney biopsy tissues from the IgAN (N = 12), control (N = 11), MCD (N = 3) and MN (N = 3) groups stained with primary antibodies for enzymes related to glycine metabolism. The stained intensities for T protein and H protein were lower in the IgAN patients than in the controls. The scale bars indicate 100 μm. MCD = minimal change disease, MN = membranous nephropathy. *P < .05, **P < .01

As glycine is primarily metabolized via the glycine cleavage system, which consists of the T, P, L and H proteins, and via serine hydroxymethyltransferase (SHMT) 1 and 2, we performed immunohistochemistry for these enzymes. The enzymes were mainly detected in kidney tubules (Figure 3). Regarding the glycine cleavage system, the protein expression levels of T and H proteins were significantly reduced in the tubulointerstitium of the IgAN patients compared to the controls. However, the expression of the other components of the glycine cleavage system or the expression of SHMT1 or SHMT2 was not significantly different in the IgAN patients.

### 3.6 In vitro experiment

**FIGURE 4:**
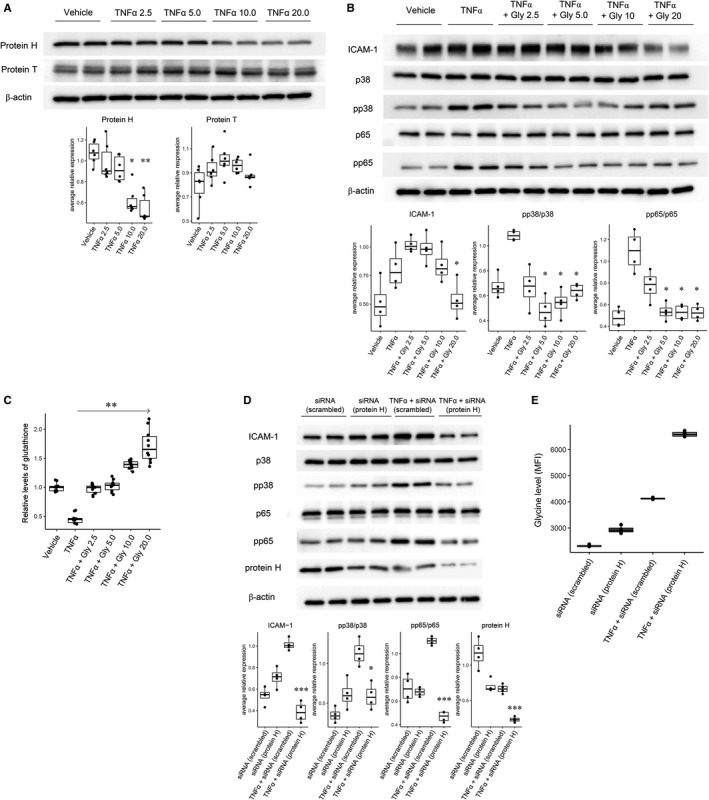
A, Representative Western blot results showing the expression of protein H and protein T in the hTECs treated with TNF‐α. Expression of protein H and T according to treatment with TNFα at different dosages (2.5, 5.0, 10.0 and 20.0 ng/mL) for 1 h. The experiment was performed 6 times, and a representative image is shown. Treatment with TNFα at 10 ng/mL and 20 ng/mL resulted in significantly reduced expression of protein H. The experiment was performed in triplicate, and a representative image is shown. *P < .05, **P < .01. B, Representative Western blot results showing inflammatory molecule expression according to the addition of glycine and TNF‐α induction in hTECs. Expression of inflammation‐related molecules according to the addition of glycine at different dosages (2.5 mmol/L, 5.0 mmol/L, 10.0 mmol/L and 20 mmol/L) in the cells treated with 10 ng/mL TNFα for 1 h. The addition of glycine resulted in a significant reduction in the expression levels of ICAM‐1, pp38 and pp65 compared to the results with only TNFα. The experiment was performed in quadruplicate, and a representative image is shown. *P < .05. C, Glutathione assay results. The results showed that the glutathione concentrations were increased along with the addition of glycine, reported in relative levels compared to the mean level of the control cells. Glutathione assay was performed for ten times in each condition. ** *P* < .01. D, Representative Western blot results of the inflammatory molecules and glycine levels according to TNF‐α induction and protein H suppression by siRNA. The suppression of protein H by siRNA under TNFα treatment resulted in a significant reduction in ICAM‐1, pp38/p38, pp65/p65 and protein H compared to the results from treatment with TNFα alone (*P* < .05, **P < .01 and *** *P* < .001). E, The glycine levels (MFI = mean fluorescent intensity) of the cell lysates were significantly increased following siRNA suppression compared to the result with scrambled RNA. Moreover, TNFα induction resulted in a further increase in glycine levels. Last, when protein H was suppressed by siRNA, the glycine levels were even higher than those in the other experiments. The experiment was performed in quadruplicate, and a representative image is shown

We found that the expression of protein H of the glycine cleavage system was significantly reduced in the hTECs treated with TNF‐α at 10.0 ng/mL or 20 ng/mL for 1 hour (Figure 4A). However, protein T expression did not show a significant change with TNF‐α treatment.

The protein expression of ICAM‐1, pp38 and pp65 was significantly increased in the hTECs treated with 10.0 ng/mL TNF‐α (Figure 4B). Moreover, the expression of pp38 and pp65 was significantly ameliorated by the addition of glycine at 5.0 mmol/L or higher. The expression of ICAM‐1 was also significantly reduced with the cotreatment of glycine at 20.0 mmol/L. In addition, the addition of glycine was significantly associated with higher glutathione levels (Figure 4C).

We next inhibited protein H via the transduction of hTECs with protein H siRNA to explore whether alteration of glycine metabolism through inhibiting the target molecules may replicate the protective effect of high glycine on inflammation in hTECs. Inhibition of protein H by siRNA significantly decreased the endogenous expression of protein H (Figure 4D), which accompanied an increase in glycine levels in the cell lysates. Treatment of hTECs with TNF‐α also resulted in reduced expression of protein H and increased glycine levels in cell lysates. When protein H was inhibited by siRNA with TNF‐α induction, the expression of protein H was further reduced, with a prominent increase in cellular glycine levels (Figure 4E). The inhibition of protein H by siRNA in the TNF‐α‐induced hTECs attenuated the expression of ICAM‐1, pp38 and pp65 compared to that of the scrambled siRNA‐transfected cells treated with TNF‐α.

## 4 DISCUSSION

In this study, we found that glycine levels were increased in the urine of IgAN patients without reduced eGFR compared to those of healthy individuals or patients with MN, MCD, or LN. When assessing the predictive power, measuring urine glycine level showed certain diagnostic value for IgAN. Moreover, higher urinary glycine levels, along with several other metabolites, were significantly associated with the prognosis of early IgAN patients, and the inclusion of urinary glycine levels significantly improved the power of a prognostic prediction model. In the glycine cleavage system, protein H expression was decreased in the tubules of the IgAN patients. Furthermore, we showed that glycine may have a protective role against inflammatory signals by in vitro experiments using hTECs. Inhibition of protein H resulted in an increase in cellular glycine levels along with amelioration of inflammatory signals. Our results suggested that urinary glycine may be a prognostic biomarker for IgAN in those without established kidney function impairment, and glycine metabolism may be a targetable pathway that can improve inflammatory insult in kidney tubules.

Although IgAN is defined as a single type of primary glomerulonephritis, the diagnosis mostly depends on morphological changes in kidney pathology, and thus, patients have heterogeneous characteristics and prognosis. Thus, efforts have been made to predict the prognosis of IgAN, and a combination of pathologic grading and clinical characteristics has been used to develop a predictive model with a certain degree of power.^4^ However, further identification of disease‐related prognostic biomarkers that can be non‐invasively collected, which may be utilized before the establishment of reduced GFR, is warranted. In this study, we performed a metabolomic screening of urinary metabolites in IgAN patients and identified that urinary glycine was significantly increased in IgAN patients without reduced eGFR compared to controls without kidney disease or those with MN, MCD, or LN. In addition, our ROC analysis indicated that although measuring diverse metabolite levels may be preferred, if a single urine metabolite is to be measured, glycine may be prioritized. This is because the model including urine glycine level showed comparable predictability to the model including all other urine metabolites and showed better predictability than the base clinical model. Furthermore, the addition of glycine or other urinary metabolites to a clinical model showed non‐inferior predictability for the prediction of early outcome in IgAN patients compared to that of the full model including pathologic characteristics, suggesting the prognostic value of non‐invasively measured biomarkers. Moreover, the addition of urinary glycine levels or other metabolites improved the full prediction model, including clinical and pathologic characteristics^4^; thus, the additive value to the current prediction tool was shown. In summary, this study suggests that the urinary glycine level may have significant diagnostic and prognostic value in early IgAN patients.

Glycine is a simple amino acid that is present at approximately 170‐330 µmol/L in human or animal blood,^16^, ^17^, ^18^ and its levels increase with dietary uptake. Intracellular levels of glycine were reported to be much higher than that in blood, and exceptionally high glycine concentrations, up to 20 mmol/L, were identified in the kidney tubules of rabbits.^19^ The major portion of glycine is taken up and metabolized in cells, and a small portion is urinarily excreted. In cells, glycine is mainly metabolized by mitochondrial and cytosolic SHMT and the glycine cleavage system, forming serine from glycine. The glycine cleavage system consists of four proteins, and the H protein mediates the intermediate reactions and modulates the active sites of other enzymes.^20^ The protective role of glycine in the kidney has been highlighted in previous studies. In particular, studies have focused on the mechanism of ischaemia‐reperfusion injury, and several studies have suggested that glycine protects kidney tubules from ischaemic injury both by *in vivo* and *in vitro* experiments.^21^, ^22^, ^23^ Furthermore, glycine showed a protective effect in transplantation organ storage and perfusion.^24^, ^25^ Even some clinical trials showed that glycine administration was associated with improved liver function parameters after liver transplantation, and a trial showed that glycine administration accelerated urinary uric acid excretion.^26^, ^27^ Regarding human kidney prognosis, urinary glycine levels have been reported to be associated with a lower risk of incident chronic kidney disease in the Framingham Offspring cohort.^15^

In the current study, we first reported the possibility of a protective role of glycine in the prognosis of early IgAN, which is the most common primary glomerulonephritis worldwide.^1^ The protective role of glycine on inflammatory signalling in hTECs was reidentified in this study, and this may be related to antioxidative effect considering the findings of altered glutathione concentration along with glycine supplementation. In addition to previous studies reporting a protective role against inflammation in other cell types, this study suggests that a high concentration of glycine may ameliorate inflammatory injury in hTECs induced by TNF‐α, which activates tubules in IgAN by glomerulotubular communication.^28^ Further, the study demonstrated that protein H of the glycine cleavage system was suppressed in IgAN patients without reduced eGFR or in hTECs induced by TNF‐α, suggesting that kidney tubules may physiologically increase glycine levels by changing their metabolism. This hypothesis implies that kidneys in patients with early IgAN fail to respond to inflammatory injury by increasing glycine levels, which are reflected in the urinary glycine concentration, and thus, these patients may have a worse prognosis than those with higher urine glycine levels. As suppression of protein H in the *in vitro* experiment also resulted in amelioration of inflammatory signals in hTECs, glycine may be a targetable metabolite that is associated with prognosis of IgAN.

Our study have several limitations and unanswered questions. First, there were other metabolites, mostly the levels that showed a strong correlation with the levels of glycine, that were associated with the prognosis of IgAN. A future study is warranted to reveal whether they are consequences of altered glycine metabolism or have an independent role in IgAN pathophysiology. Second, although glycine was higher in the IgAN group than in both the healthy control group and the MN/MCD/LN groups, this finding does not mean that the role of glycine is IgAN specific. Given previous reports suggesting various benefits of glycine in ischaemic‐reperfusion injury or inflammatory conditions, the protective role of glycine in the kidney may be due to general mechanisms. This study suggests that glycine can be a prioritized metabolite in early IgAN as the metabolism of glycine was significantly altered in IgAN without prominently reduced eGFR. Third, additional external validation of the results in an independent cohort or a repetitive measurement of urine metabolites during the follow‐up period is necessary to confirm the possible diagnostic or prognostic value of urinary glycine. Despite the relatively large number of IgAN patients, a selection bias remains in this study. Fourth, additional studies are warranted to investigate the possible benefits of interventions targeting glycine in human IgAN. As glycine is an amino acid normally ingested in foods and generally tolerated in humans, additional supplementation with glycine may be considered. However, this study only suggests the possibility but cannot confirm whether increasing the external intake of glycine or targeting the glycine metabolic system can replicate the possible benefits of glycine in human IgAN that was shown in hTECs. Last, our findings may not be applicable to patients with reduced eGFR, as all the study patients had preserved eGFR.

In conclusion, urinary glycine levels were increased in IgAN patients without reduced eGFR compared to healthy, MN, MCD, and LN controls. Higher levels of urinary glycine were associated with better prognosis of early IgAN, and the addition of the urine metabolite to the prognostic prediction model of IgAN resulted in improved predictive power. As glycine and related metabolic pathways may have protective roles against inflammatory injury in hTECs, a future study may target glycine when considering interventions to improve the prognosis of IgAN.

## CONFLICT OF INTEREST

The authors declare no conflicts of interest.

## AUTHOR CONTRIBUTIONS

**Sehoon Park:** Conceptualization (equal); Data curation (equal); Formal analysis (equal); Funding acquisition (equal); Methodology (equal); Project administration (equal); Resources (equal); Software (equal); Writing‐original draft (lead); Writing‐review & editing (equal). **Jueun Lee:** Conceptualization (equal); Data curation (lead); Formal analysis (equal); Investigation (equal); Methodology (lead); Project administration (equal); Resources (equal); Software (equal); Visualization (equal); Writing‐original draft (lead); Writing‐review & editing (equal). **Seung Hee Yang:** Data curation (equal); Formal analysis (equal); Funding acquisition (equal); Investigation (lead); Methodology (lead); Resources (equal); Software (equal); Supervision (equal); Validation (equal); Visualization (lead); Writing‐original draft (lead); Writing‐review & editing (equal). **Hajeong Lee:** Data curation (equal); Formal analysis (equal); Investigation (equal); Resources (equal); Software (equal); Supervision (equal); Writing‐original draft (equal). **Joo Young Kim:** Data curation (equal); Formal analysis (equal); Investigation (equal); Methodology (equal); Resources (equal); Writing‐original draft (equal). **Minkyoung Park:** Data curation (equal); Formal analysis (equal); Investigation (equal); Methodology (equal); Project administration (equal); Visualization (equal); Writing‐original draft (equal). **Kyu Hong Kim:** Data curation (equal); Formal analysis (equal); Methodology (equal); Supervision (equal); Validation (equal); Writing‐original draft (equal). **Jong Joo Moon:** Conceptualization (equal); Data curation (equal); Investigation (equal); Methodology (equal); Visualization (equal); Writing‐original draft (equal). **Semin Cho:** Investigation (equal); Methodology (equal); Validation (equal); Visualization (equal); Writing‐original draft (equal). **Soojin Lee:** Data curation (equal); Formal analysis (equal); Methodology (equal); Project administration (equal); Visualization (equal); Writing‐original draft (equal). **Yaerim Kim:** Data curation (equal); Investigation (equal); Methodology (equal); Visualization (equal); Writing‐review & editing (equal). **Hajeong Lee:** Data curation (equal); Funding acquisition (equal); Investigation (equal); Project administration (equal); Supervision (equal); Validation (equal); Writing‐review & editing (equal). **Jung Pyo Lee:** Data curation (equal); Formal analysis (equal); Methodology (equal); Resources (equal); Software (equal); Writing‐review & editing (equal). **Chang Wook Jeong:** Data curation (equal); Formal analysis (equal); Investigation (equal); Methodology (equal); Supervision (equal); Writing‐review & editing (equal). **Cheol Kwak:** Data curation (equal); Formal analysis (equal); Methodology (equal); Supervision (equal); Writing‐review & editing (equal). **Kwon Wook Joo:** Conceptualization (equal); Data curation (equal); Investigation (equal); Methodology (equal); Writing‐review & editing (equal). **Chun Soo Lim:** Formal analysis (equal); Investigation (equal); Methodology (equal); Software (equal); Supervision (equal); Writing‐review & editing (equal). **Yon Su Kim:** Data curation (equal); Formal analysis (equal); Investigation (equal); Methodology (equal); Resources (equal); Supervision (equal); Validation (equal); Visualization (equal); Writing‐review & editing (equal). **Geum‐Sook Hwang:** Data curation (equal); Formal analysis (equal); Funding acquisition (equal); Investigation (equal); Methodology (equal); Software (equal); Supervision (equal); Validation (equal); Visualization (equal); Writing‐original draft (equal); Writing‐review & editing (lead). **Dong Ki Kim:** Conceptualization (equal); Data curation (equal); Formal analysis (equal); Funding acquisition (lead); Investigation (equal); Methodology (lead); Project administration (equal); Resources (equal); Software (equal); Supervision (equal); Validation (equal); Visualization (equal); Writing‐original draft (lead); Writing‐review & editing (lead).

## Supporting information

Supplementary Material

## Data Availability

The data for this study are available from the corresponding author under reasonable request.
