# Supplementary material for: Comprehensive metabolomic profiling in early IgA nephropathy patients reveals urine glycine as a prognostic biomarker
Source: J Cell Mol Med. 2021 May 3;25(11):5177–90. doi: 10.1111/jcmm.16520 (PMC8178259; doi:10.1111/jcmm.16520)

Supplemental Material

Comprehensive metabolomic profiling in early IgA nephropathy patients reveals urine glycine as a prognostic biomarker

Authors

*Sehoon Park, MD,[1,2] *Jueun Lee, PhD,[3] *Seung Hee Yang, PhD,[4] Hajeong Lee,[3] Joo Young Kim,[4] Minkoung Park,[4] Kyu Hong Kim,[4] Jong Joo Moon, MD,[5] Semin Cho, MD,[5] Soojin Lee, MD,[5] Yaerim Kim, MD, PhD,[6] Hajeong Lee, MD, PhD,[5,7] Jung Pyo Lee, MD, PhD,[4,7,8] Kwon Wook Joo, MD, PhD,[4,5,7] Chun Soo Lim, MD, PhD,[4,5,8] Yon Su Kim, MD, PhD,[1,4,5,7] #Geum-Sook Hwang, PhD,[3,9] and #Dong Ki Kim, MD, PhD[4,5,7]

Authors' affiliations

[1]Department of Biomedical Sciences, Seoul National University College of Medicine, Seoul, Korea

[2]Department of Internal Medicine, Armed Forces Capital Hospital, Gyeonggi-do, Korea

[3]Integrated Metabolomics Research Group, Western Seoul Center, Korea Basic Science Institute, Seoul, Korea

[4]Kidney Research Institute, Seoul National University, Seoul, Korea

[5]Department of Internal Medicine, Seoul National University Hospital, Seoul, Korea

[6]Department of Internal Medicine, Keimyung University School of Medicine, Daegu, Korea

[7]Department of Internal Medicine, Seoul National University College of Medicine, Seoul, Korea

[8]Department of Internal Medicine, Seoul National University Boramae Medical Center, Seoul, Korea

[9]Department of Chemistry and Nano Science, Ewha Womans University, Seoul, Korea.

*These authors contributed equally to this work.

#Co-correspondence

Table of Contents

Supplemental Methods.

Supplemental Table 1. Creatinine-adjusted urine metabolite levels.
Supplemental Table 2. Association between the metabolite levels and the risk of eGFR 30% reduction.

Supplemental Figure 1. A representative 800 MHz ^{1}H NMR spectrum of urine from a IgAN patient with metabolite assignments.
Supplemental Figure 2. Representative single reaction ion chromatograms of glycine in a urine sample from IgAN patient (A) and a standard sample (B). A standard curve plot for glycine (C).
Supplemental Figure 3. Plots visualizing the principal component analysis and the partial least squares discriminant analysis results.
Supplemental Figure 4. Correlation between the clinical characteristics of IgAN patients and the urine metabolite levels.
Supplemental Figure 5. Correlation between urine metabolites.
Supplemental Figure 6. Internal validation results with the subsamples of IgAN patients comparing the urine glycine levels measured by NMR method and LC-MS method.

Supplemental Methods

Methods for collected clinical data

Baseline characteristics, including age, sex, baseline eGFR, mean arterial pressure (calculated from 1/3 systolic BP plus 2/3 systolic BP), baseline urine protein-to-creatinine ratio, and other laboratory findings, were collected. For IgAN patients, information on the history of medication usage, including that of angiotensin-converting enzyme inhibitor or angiotensin II receptor blocker and immunosuppressive drugs, was collected. The Oxford classification was scored to reflect the pathological grades.

Methods for NMR-based urine metabolomic profiling

The collected urine samples were immediately stored at 4 °C. Within a few hours, we centrifuged the collected urine samples at 2,500 rpm at 4 °C for 10 min, and the supernatants were frozen at -80 °C for later metabolomic analysis by NMR spectrometry. In brief, proteins in the urine samples were removed with centrifugal filters (Amicon Ultra, 3K, Merck Millipore, USA) at 12,700 rpm and 4 °C for 15 min. Then, 300 μL of filtered urine was mixed with 330 μL of 0.2 M sodium phosphate buffer (pH 7.0) and 70 μL of 5 mM 3-(trimethylsilyl) propionic 2,2,3,3-d4 acid sodium salt (TSP, 98 atom %). After mixing, 600 μL of the sample was transferred into a 5-mm NMR tube. One-dimensional (1D) ^{1}H NMR spectra of urine were acquired with 64 transients at 298 K using a Bruker Avance III HD 800-MHz NMR spectrometer (Bruker BioSpin, Germany) equipped with a Bruker 5 mm CPTCI Z-GRD probe using a NOESYPRESAT pulse sequence. All acquired ^{1}H NMR spectra were phased and baseline-corrected using TopSpin 3.1 and AMIX (Bruker BioSpin). The processed NMR spectra were imported into Chenomx (version 7.1, Edmonton, AB, Canada) for identification and quantification of the urine metabolomes. Identification of the urine metabolome was performed using the 800 MHz library of Chenomx, 2D NMR spectra and spiking experiments. A representative ^{1}H NMR spectrum of urine from an IgAN patient is shown in Supplemental Figure 1. The levels of urinary metabolites were quantified by integrating peak areas of metabolites compared with the areas of the TSP peak. The final reported 26 urinary metabolite levels were additionally adjusted with urine creatinine levels.

Next, we internally validated the measured amount of glycine with liquid chromatography-mass spectrometry (LC-MS). Technical replicates from subsamples of the IgAN patients were used for this internal validation. Briefly, 30 μL of urine was mixed with 90 μL of cold methanol for 1 min, kept at -20 °C for 30 min to complete protein precipitation, and

centrifuged at 12,700 rpm for 20 min at 4 °C. Then, the supernatant was transferred to a new 1.5 mL tube and dried. The pellet was resolved with 300 μL of 20% aqueous methanol (v/v) and vortexed for 1 min. After dilution with 20% aqueous methanol (v/v) 10 or 100 times, urine extracts were mixed with 20 μL of internal standard (serine-$^{13}C_3$, 1 mg/mL). Finally, 2 μL was injected into the LC-MS system, and an Agilent 1290 Infinity LC and Agilent 6495 Triple Quadrupole MS system equipped with an Agilent Jet Stream ESI source (Agilent Technologies, USA) was applied to measure the glycine concentration. The urine samples were eluted and separated using a Scherzo SM-C18 column (100 x 2 mm, 3 μm, Imtakt, USA) for 15 min at 25 °C. The mobile phase for gradient elution consisted of water and methanol containing formic acid (0.1%, v/v, eluents A and B, respectively). The flow rate was set to 0.2 mL/min using the following gradient: 0–3 min of 100% A, 3–7 min to 0% A, 7–10 min hold at 0% A, 10–11 min at 100% A and 11–15 min hold at 100% A. MS/MS experiments were conducted in positive ion mode with the following parameters: capillary voltage of 3.5 kV, nebulizer gas of nitrogen at 40 psi, drying gas temperature of 120 °C, drying gas flow rate of 11 L/min, sheath gas temperature of 350 °C and sheath gas flow rate of 12 L/min. The ion transition selected for MS/MS detection was m/z 76.1 → 30.1 for glycine with a collision energy of 8.0 V. Supplemental Figure 2 shows representative extract ion chromatograms of glycine in urine from the IgAN patients and standards and a standard curve for quantification. Finally, the glycine concentration was adjusted with urine creatinine levels, which were assessed following the manufacturer's instructions (Abcam, Cambridge, UK, Cat No. ab65340).

Supplemental Table 1. Creatinine-adjusted urine metabolite levels.

	IgAN (N=201)	MN (N=77)	MCD (N=47)	LN (N=36)	Control (N=136)	IgAN vs. MN P value	IgAN vs. MCD P value	IgAN vs. LN P value	IgAN vs. Control P value
1-Methylnicotinamide	4.4 [2.7; 7.5]	3.8 [2.6; 5.5]	5.2 [2.7; 7.5]	5.2 [3.2; 7.9]	6.3 [3.9;11.1]	0.109	0.923	0.109	< 0.001
2-Hydroxyisobutyrate	7.3 [5.7; 8.8]	7.5 [5.9; 9.3]	7.2 [5.2; 9.4]	6.6 [4.6; 8.3]	7.2 [5.6; 9.4]	0.535	0.819	0.535	0.72
3-Indoxylsulfate	18.6 [10.2;30.4]	11.2 [3.5;18.9]	9.9 [2.4;26.3]	18.5 [10.3;31.9]	18.7 [11.3;26.8]	< 0.001	0.011	< 0.001	0.953
Acetate	5.1 [3.4; 8.3]	6.3 [3.5; 9.7]	4.8 [3.0; 7.7]	7.7 [4.3;10.4]	4.6 [3.2; 7.0]	0.055	0.51	0.055	0.191
Acetone	1.6 [0.9; 2.9]	2.3 [1.4; 3.9]	1.8 [1.4; 2.7]	2.2 [1.4; 3.3]	2.2 [1.4; 3.8]	0.013	0.195	0.013	< 0.001
Alanine	37.3 [28.3;51.0]	41.4 [27.9;64.4]	40.0 [23.5;53.8]	33.4 [25.2;42.9]	25.4 [17.1;34.1]	0.095	0.925	0.095	< 0.001
Betaine	15.4 [9.8;24.8]	17.3 [11.8;37.2]	16.3 [11.6;28.0]	10.1 [7.8;18.1]	10.9 [7.0;15.2]	0.04	0.304	0.04	< 0.001
Choline	4.1 [2.5; 6.9]	6.2 [3.8;11.0]	6.1 [3.2; 9.2]	5.3 [3.2; 8.4]	2.3 [1.6; 3.5]	< 0.001	0.009	< 0.001	< 0.001
Citrate	303.1 [185.4;500.5]	369.1 [205.8;542.6]	234.9 [131.0;357.7]	94.3 [50.8;191.6]	321.4 [177.3;578.7]	0.196	0.014	0.196	0.477
Dimethylamine	44.2 [37.5;56.4]	52.0 [41.7;61.7]	45.3 [39.6;60.1]	57.7 [47.8;69.0]	38.3 [31.7;50.5]	0.017	0.441	0.017	< 0.001
Formate	22.0 [14.2;29.9]	25.2 [16.6;38.6]	21.3 [12.5;31.0]	18.8 [12.0;34.5]	17.0 [9.1;26.0]	0.019	0.726	0.019	0.001
Fumarate	0.3 [0.0; 0.5]	0.4 [0.0; 1.0]	0.4 [0.0; 0.6]	0.4 [0.0; 0.7]	0.3 [0.2; 0.4]	0.011	0.328	0.011	0.313
Glucose	48.5 [36.4;63.1]	70.0 [49.2;145.9]	57.2 [45.4;70.8]	52.4 [38.4;73.8]	49.5 [39.3;68.2]	< 0.001	0.03	< 0.001	0.388
Glycine	124.9 [83.0;191.8]	91.2 [70.2;129.2]	75.1 [53.9;96.6]	80.8 [58.0;102.5]	74.9 [48.7;113.0]	0.001	< 0.001	0.001	< 0.001
Isoleucine	1.8 [1.4; 2.2]	2.3 [1.7; 3.3]	2.2 [1.8; 2.9]	2.1 [1.4; 2.9]	1.4 [1.1; 1.6]	< 0.001	0.001	< 0.001	< 0.001
Lactate	13.8 [8.6;21.6]	13.6 [9.5;20.9]	12.8 [8.6;20.3]	16.0 [11.2;23.6]	9.6 [6.9;13.2]	0.849	0.419	0.849	< 0.001
Leucine	4.3 [3.5; 5.6]	6.0 [4.2; 7.3]	5.7 [4.7; 7.2]	4.8 [3.9; 7.1]	3.1 [2.4; 3.8]	< 0.001	< 0.001	< 0.001	< 0.001
N,N-Dimethylglycine	6.0 [4.2; 8.8]	6.3 [3.6; 9.6]	6.0 [3.5; 7.9]	4.3 [2.1; 7.0]	4.4 [2.8; 6.3]	0.849	0.572	0.849	< 0.001
Phenylalanine	34.8 [17.8;61.8]	34.8 [13.5;66.5]	26.9 [13.0;60.8]	50.3 [25.2;90.6]	26.4 [13.1;56.7]	0.775	0.485	0.775	0.104
Pyruvate	3.4 [2.3; 4.6]	3.3 [2.4; 4.5]	3.4 [2.5; 4.6]	3.3 [2.2; 5.0]	2.0 [1.2; 2.8]	0.804	0.765	0.804	< 0.001
Taurine	160.6 [107.3;251.1]	188.3 [136.1;258.1]	193.2 [108.0;263.2]	147.2 [97.1;212.9]	157.7 [112.4;219.9]	0.094	0.491	0.094	0.684
Threonine	18.1 [13.8;25.6]	19.2 [14.3;27.3]	19.7 [13.7;25.7]	16.7 [12.9;24.7]	12.8 [9.4;18.3]	0.315	0.516	0.315	< 0.001
TrimethylamineN-oxide	78.7 [43.6;172.3]	92.5 [46.4;264.6]	90.7 [44.9;172.8]	121.5 [59.8;243.0]	47.1 [26.3;112.3]	0.282	0.869	0.282	< 0.001
Tryptophan	7.3 [5.9;10.2]	8.4 [5.5;10.7]	8.2 [5.6;11.1]	6.6 [4.1;10.1]	7.1 [5.2; 8.9]	0.532	0.298	0.532	0.04
Valine	5.3 [4.2; 6.2]	6.9 [5.3; 8.7]	6.4 [5.0; 7.3]	5.5 [4.6; 7.4]	3.9 [3.2; 4.6]	< 0.001	0.002	< 0.001	< 0.001
τ-Methylhistidine	25.7 [22.0;30.6]	24.8 [21.5;30.2]	24.6 [19.8;28.7]	25.4 [22.8;30.2]	20.4 [17.4;24.7]	0.6	0.219	0.6	< 0.001

Supplemental Table 2. Association between the metabolite levels and the risk of eGFR 30% reduction.

	Univariable model		Multivariable model†	
	sHR (95% CI)	P	Adjusted sHR (95% CI)	P
Alanine	0.63 (0.40-0.98)	0.04	0.50 (0.29-0.86)	0.01
Betaine	1.01 (0.75-1.37)	0.93	0.96 (0.67-1.38)	0.82
Citrate	0.56 (0.36-0.88)	0.01	0.62 (0.37-1.04)	0.07
Dimethylamine	0.75 (0.47-1.18)	0.21	0.67 (0.38-1.19)	0.17
Glucose	0.96 (0.65-1.43)	0.85	0.69 (0.36-1.33)	0.27
Glycine	0.49 (0.30-0.82)	0.006	0.44 (0.24-0.83)	0.01
Threonine	0.47 (0.26-0.83)	0.009	0.37 (1.19-0.72)	0.003
Valine	0.74 (0.50-1.11)	0.15	0.52 (0.32-0.84)	0.008

All metabolite values were included in the Cox regression models as continuous variables; 1 standard deviation increment. The calculated standardized hazard ratios (sHR) with 95% confidence intervals (CI) are presented.

Among 201 IgAN patients, 37 patients experienced eGFR 30% reduction.

†Multivariable model was adjusted for age, sex, baseline eGFR, mean arterial pressure, urine protein-to-creatinine ratio, MEST-C pathologic scores, and history of angiotensin-converting enzyme inhibitor or angiotensin II receptor blocker, and history of immunosuppressive agent at the time of kidney biopsy.

Supplemental Figure 1. A representative 800 MHz ^{1}H NMR spectrum of urine from a IgAN patient with metabolite assignments.

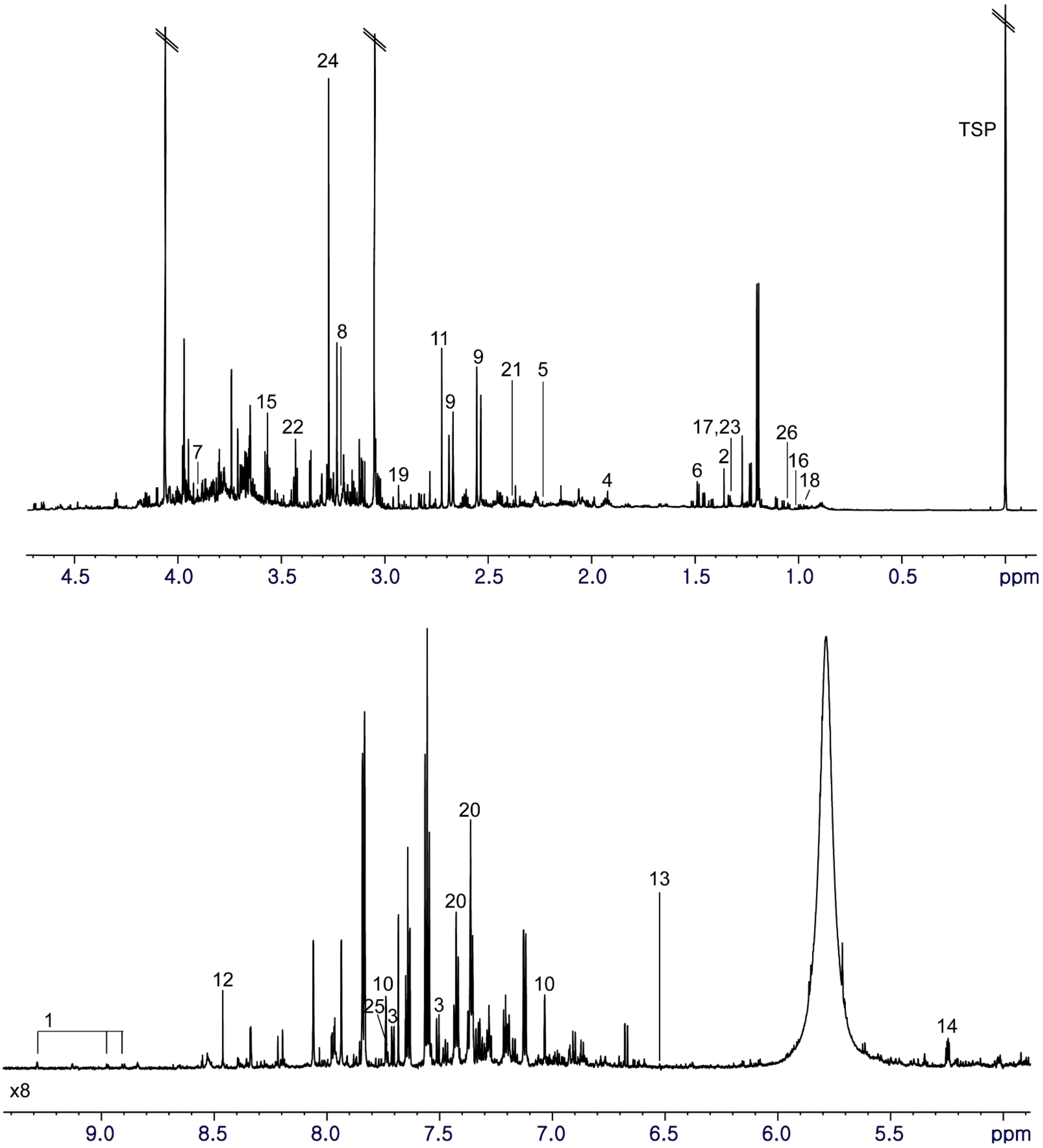

1. 1-methylnicotinamide; 2. 2-hydroxyisobutyrate; 3. 3-indoxylsulfate; 4. acetate; 5. acetone; 6. alanine; 7. betaine; 8. choline; 9. citrate; 10. τ-methylhistidine; 11. dimethylamine; 12. formate; 13. fumarate; 14. glucose; 15. glycine; 16. isoleucine; 17. lactate; 18. leucine; 19. N,N-dimethylglycine; 20. phenylalanine; 21. pyruvate; 22. taurine; 23. threonine; 24. trimethylamine N-oxide; 25. tryptophan; 26. valine.

Supplemental Figure 2. Representative single reaction ion chromatograms of glycine in a urine sample from IgAN patient (A) and a standard sample (B). A standard curve plot for glycine (C).

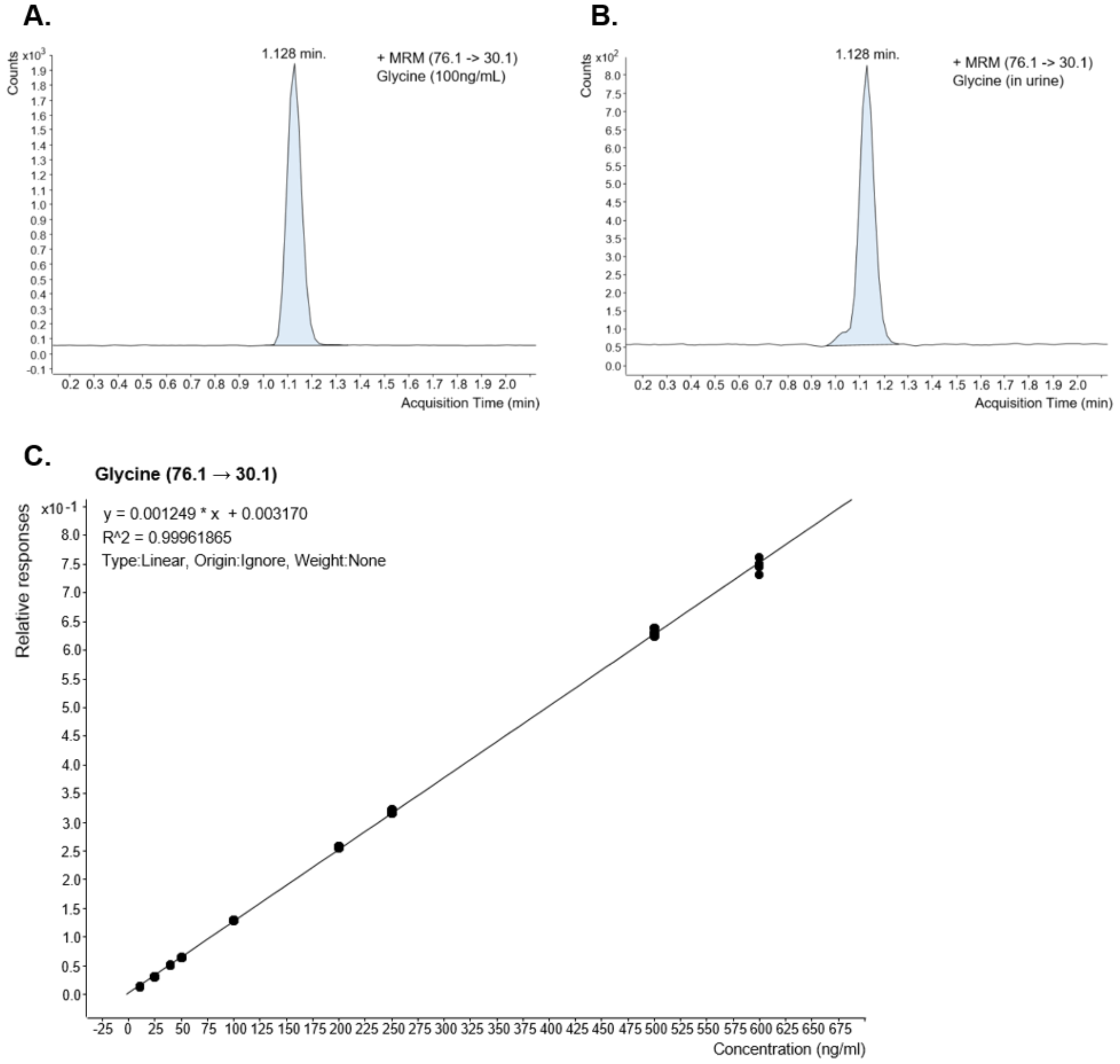

Supplemental Figure 3. Plots visualizing the principal component analysis and the partial least squares discriminant analysis results.

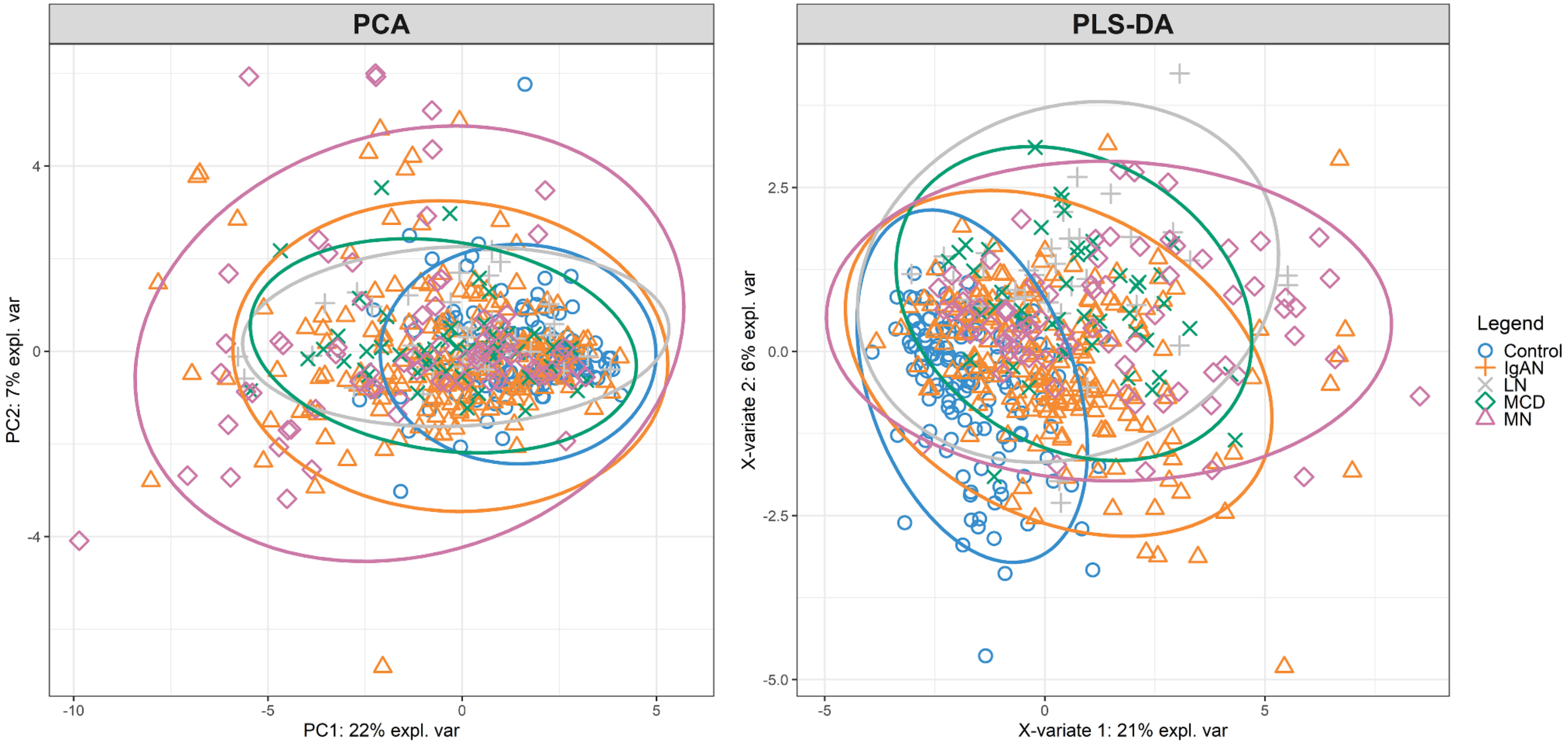

The principal component analysis plot with first and the second principal components is presented in the left. The partial least squares discriminant analysis plot showing the first and the second variates explaining the total variance are presented in the right. The legend indicating the IgAN, MN, MCD, LN, and healthy control group is presented in the right side of the graph. PCA = principal component analysis, PLS-DA = partial least squares discriminant analysis

Supplemental Figure 4. Correlation between the clinical characteristics of IgAN patients and the urine metabolite levels.

	Age	eGFR	UPCR	MAP	Albumin	Diabetes	M	E	S	T	C
1-Methylnicotinamide	0.15	0.06	-0.03	-0.1	-0.06	0.03	-0.11	0	0.04	-0.03	0.07
2-Hydroxyisobutyrate	0.2	0.02	0.04	-0.06	0.02	0.12	-0.11	-0.02	0.09	-0.06	0.01
3-Indoxylsulfate	-0.04	0.05	-0.15	-0.06	0	-0.14	0.02	-0.03	0.01	-0.06	-0.05
Acetate	0.31	-0.06	0.03	-0.09	-0.05	0.05	-0.07	-0.12	0.05	-0.07	0.04
Acetone	0.23	-0.08	-0.01	-0.12	0.07	0.1	-0.13	-0.02	-0.06	-0.04	-0.09
Alanine	0.11	0.11	0.16	-0.04	-0.1	0.14	0	-0.07	0.11	0.05	-0.01
Betaine	0.36	-0.23	0.25	0	-0.13	0.21	-0.1	-0.02	0.16	0.08	0.09
Choline	0.33	-0.19	0.34	-0.02	-0.41	0.24	0.05	0.01	0.12	0.12	0.23
Citrate	0.23	0.09	-0.1	-0.22	0.12	0.12	-0.16	-0.06	0.01	-0.1	-0.03
Dimethylamine	0.34	-0.11	0.13	0.01	-0.2	0.02	-0.06	-0.05	0.03	0.03	0.16
Formate	0.14	0.19	0.02	-0.22	-0.02	0.05	-0.18	-0.05	-0.08	-0.11	0.01
Fumarate	0.16	-0.05	0.15	-0.12	-0.26	0.06	0.03	0	0.17	0.08	0.08
Glucose	0.25	-0.11	0.26	0.06	-0.17	0.29	0.03	-0.06	0.1	0	0.16
Glycine	-0.1	0.26	0.02	-0.19	-0.04	-0.03	-0.02	-0.03	0.09	-0.03	0.07
Hippurate	0.07	0.12	0.2	-0.11	-0.19	0.08	0.04	-0.02	0.09	0.07	0.09
Lactate	0.3	0.04	0.16	-0.28	-0.14	0.14	-0.04	-0.1	0.04	0.07	-0.06
Leucine	0.18	0.07	0.28	-0.07	-0.22	0.16	0.04	0	0.12	0.1	0.19
N,N-Dimethylglycine	0.16	-0.02	0.12	-0.01	-0.08	0.06	-0.02	-0.03	0.13	0	0.1
Phenylalanine	0.04	0	-0.03	-0.12	-0.11	-0.12	0	-0.03	0	-0.08	0.01
Pyruvate	0.05	0.09	0.18	-0.12	-0.12	0.14	0	0.07	-0.04	0.07	0.06
Taurine	0.1	0.02	-0.11	-0.11	0.11	-0.02	-0.16	0.02	0.01	-0.17	-0.04
Threonine	-0.08	0.26	0.06	-0.09	-0.02	0.07	-0.01	-0.04	0.06	-0.04	0.06
Trimethylamine N-oxide	0.28	-0.12	0.04	0.04	-0.05	0.05	-0.03	0.02	-0.06	-0.08	0.11
Tryptophan	0.02	0.11	-0.01	0.05	-0.09	-0.01	-0.07	-0.03	0.06	-0.04	0.04
Valine	0.14	0.16	0.22	-0.12	-0.11	0.17	0	-0.04	0.09	0.05	0.09
τ-Methylhistidine	-0.01	0.07	0	-0.13	-0.03	-0.04	-0.13	-0.13	0.06	-0.01	0

The correlation analysis was done with the Spearman's test as the variables included ordinal Oxford classification. The correlation coefficients between the variables in rows and columns are shown, and the Bonferroni significant [$P < 0.05/(26\times11)$] correlations are colored, with red showing positive correlations and blue showing negative correlations. Albumin was serum albumin level. eGFR = estimated glomerular filtration rate, UPCR = urine protein-to-creatinine ratio, MAP = mean arterial presssure

Supplemental Figure 5. Correlation between urine metabolites.

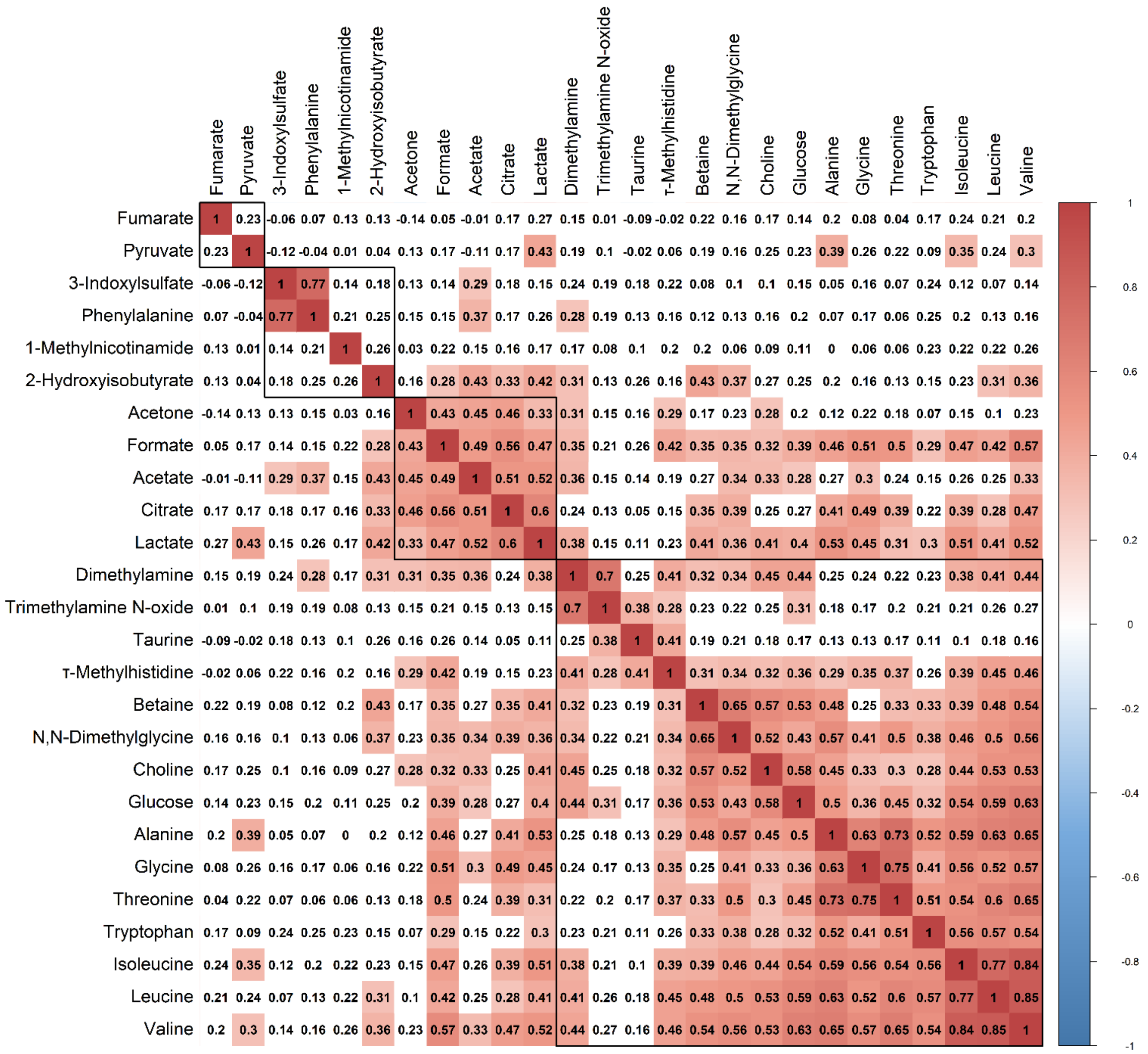

The correlation analysis was done with the Spearman’s test. The correlation coefficients between the variables in rows and columns are shown, and the significant [P < 0.05/(26×25)] correlations are colored, with red showing positive correlations and blue showing negative correlations. Hierarchal clustering was performed and the metabolites in the cluster can be identified by black square lines.

Supplemental Figure 6. Internal validation results with the subsamples of IgAN patients comparing the urine glycine levels measured by NMR method and LC-MS method.

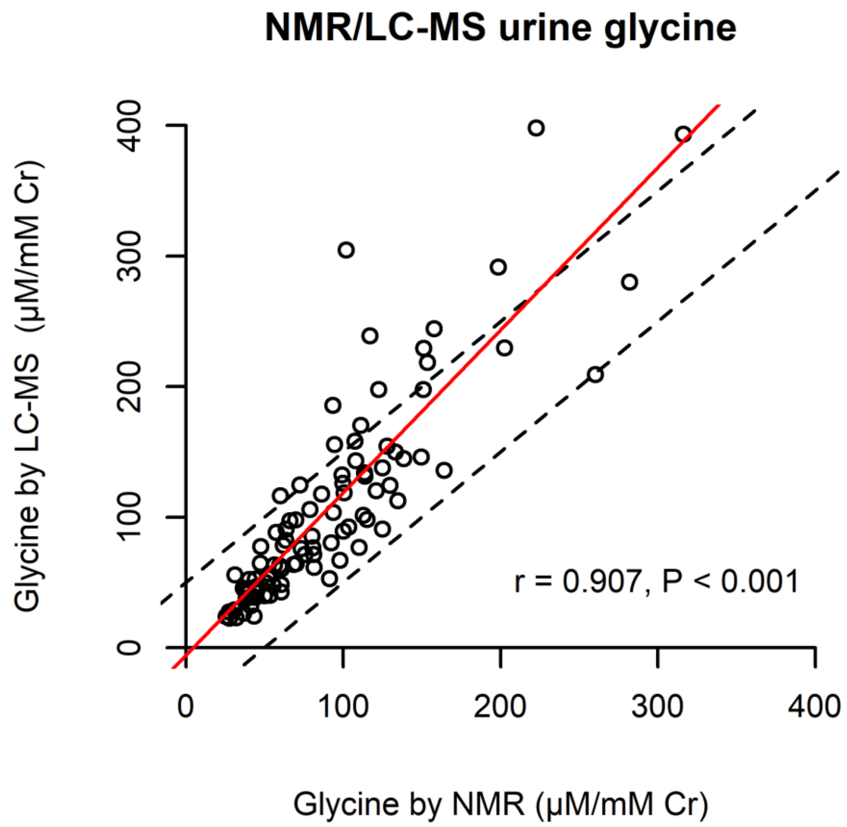

Supplement: Supplementary file 1 — Supplementary Material [file JCMM-25-5177-s001.pdf]
